# Post-operative rehabilitation using a digital healthcare system in patients who had undergone rotator cuff repair: protocol for a single-center randomized controlled trial

**DOI:** 10.1186/s13063-022-06648-4

**Published:** 2022-08-17

**Authors:** Won Kee Chang, Jong In Lee, Ji Hye Hwang, Jae-Young Lim

**Affiliations:** 1 Department of Rehabilitation Medicine, Seoul National University Bundang Hospital, Seongnam, Gyeonggi Republic of Korea; 2 Department of Rehabilitation Medicine, The Catholic University of Korea College of Medicine, Seoul, Republic of Korea; 3 Department of Physical and Rehabilitation Medicine, Sungkyunkwan University School of Medicine, Seoul, Republic of Korea; 4 Department of Rehabilitation Medicine, Seoul National University College of Medicine, Seoul, Republic of Korea

**Keywords:** Rotator cuff injuries, Postoperative care, Rehabilitation, Augmented reality

## Abstract

**Background:**

Operative repair of a rotator cuff tear requires up to 12 weeks of post-operative (post-op) home-based rehabilitation. Maintaining patients’ compliance in the post-op rehabilitation program is a pivotal component for generating successful outcomes. By developing a post-op rehabilitation-oriented digital healthcare system and applying it in patients who had undergone rotator cuff repair, we aim to increase the efficacy of the rehabilitation program and raise patients’ compliance levels. Here, we present a protocol developed for comparing the efficacy of rehabilitation using a newly developed augmented reality (AR)-based digital healthcare system with that of conventional rehabilitation for post-op rehabilitation of rotator cuff repair.

**Methods:**

This study will recruit a total of 115 patients who had undergone rotator cuff repair within 3 days after surgery. Patients will be randomly allocated to rehabilitation using an AR-based digital healthcare system (digital group) or conventional rehabilitation (conventional group). Patients in both groups will perform brochure-based exercises from the immediate post-op period to post-op 6 weeks. From post-op 6 weeks to 12 weeks, patients in the digital group will use the AR-based system for post-op exercises, whereas patients in the conventional group will continue brochure-based rehabilitation exercises. The primary outcome will be scores on the Simple Shoulder Test at post-op 12 weeks. Secondary outcomes include numeric rating scale scores for pain, measures of range of motion and muscle strength of the affected shoulder, grip strength of the affected arm, scores on the Disabilities of the Arm, Shoulder and Hand test, the Shoulder Pain and Disability Index, and the EuroQoL-5D-5L quality-of-life measure. Analyses will be conducted using an intention-to-treat approach.

**Discussion:**

This study will examine the effectiveness of an AR-based digital healthcare system for post-op rehabilitation in the patients after rotator cuff repair. The study will add evidence for the application of digital healthcare systems in post-op rehabilitation.

**Trial registration:**

ClinicalTrials.gov NCT04511377. Registered on 10 August 2020.

**Supplementary Information:**

The online version contains supplementary material available at 10.1186/s13063-022-06648-4.

## Introduction

Rotator cuff tears result in pain and dysfunction of the shoulder and are one of the most common causes of shoulder disability [1, 2]. Previous studies have estimated the prevalence of rotator cuff tears among the general population at 20% [3] to 23% [4], with a higher prevalence associated with an older age [5]. Treatment of rotator cuff tears can be divided into operative and non-operative treatments. Operative repair of the rotator cuff, which is mainly performed in patients who are unresponsive to conservative management, has demonstrated good results, with reduced pain and good functional recovery of the shoulder [6, 7].

Post-operative (post-op) rehabilitation is a pivotal component of successful rotator cuff repair. The current consensus on rehabilitation after rotator cuff repair suggests 4–6 weeks of immobilization followed by another 4–6 weeks of rehabilitation directed at recovery of range of motion (ROM) and rotator cuff muscle strengthening [8, 9]. Post-op rehabilitation thus takes up to 12 weeks after surgery and is usually performed with outpatient-based or home-based rehabilitation programs. Enhancing the efficacy of the rehabilitation program and improving patient compliance in post-op rehabilitation are important factors for achieving successful outcomes [10]. Thus, various forms of rehabilitation programs, including video instructions [11] and individualized supervision [12], have been attempted to raise patient compliance levels and increase the efficacy of rehabilitation programs.

Recent developments in information and communication technologies have led to attempts to implement virtual reality (VR)- or augmented reality (AR)-based healthcare systems in musculoskeletal post-op rehabilitation [13–15]. These VR- or AR-based rehabilitation programs have shown advantages in terms of increasing patients’ compliance levels and documenting their progress [16, 17], essential factors for successful post-op rehabilitation. However, most previous studies have used commercial healthcare applications or devices providing generalized fitness exercise programs rather than rehabilitation-oriented programs. Developing and applying an AR-based digital healthcare system with post-op rehabilitation-oriented programs for patients who had undergone rotator cuff repair would increase the efficacy of rehabilitation and improve patient compliance. In this report, we present a protocol for evaluating the efficacy of an AR-based digital healthcare system for post-op rehabilitation of rotator cuff repair by comparing it with that of a conventional rehabilitation program in an assessor-blinded 1:1 randomized controlled single-center trial.

## Methods

### Study design

**Fig. 1:**
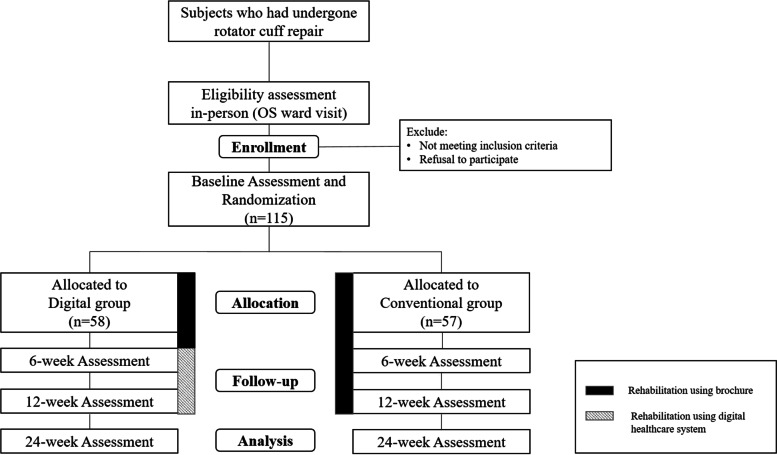
Study flow diagram

This protocol was developed for an assessor-blinded superiority randomized controlled trial. The eligible participants are recruited 1–3 days after surgery at the orthopedic ward of Seoul National University Bundang Hospital (Seongnam-si, Gyeonggi-do, Korea). The participants are then randomized based on 1:1 allocation to two groups: the digital group will undergo a rehabilitation program using a digital healthcare system, whereas the conventional group will undergo a conventional rehabilitation program. Patients will be discharged home a few days after the surgery with a shoulder brace on the affected shoulder and will immediately begin the rehabilitation program, including early mobilization of the shoulder girdle. Until post-op 6 weeks, when they will remove their braces, both groups will use brochures received at the time of discharge that provide instructions on mobilizing the affected shoulder girdle. From post-op 6 to 12 weeks, participants in the digital group will perform exercises using an AR-based digital healthcare system (UINCARE Home+; UINCARE Corp., Seoul, Korea), whereas participants in the conventional group will perform exercises using only the brochures provided by the hospital. Participants in both groups will be allowed hospital visits for exercise education by physical therapists, up to three sessions (30 min per session) over 12 weeks. The primary and secondary outcomes will be measured at baseline and at post-op 6, 12, and 24 weeks. An overview of the study design and process is given in Fig. 1.

### Participants and recruitment

In the protocol, the aim is to enroll 115 adult participants over 50 years of age who had undergone rotator cuff repair surgery. Patients who had undergone biceps tenotomy, acromioplasty, or labral repair along with the rotator cuff repair can be included. Patients who had undergone reverse total shoulder arthroplasty or total shoulder arthroplasty will be excluded. Patients who are not discharged home or who cannot understand or read Korean will also be excluded. Other major inclusion and exclusion criteria are described in Table 1. Participants will be recruited from the orthopedic surgery ward at 1–3 days after surgery by research therapists from the study team. The research team will screen the medical records of the post-op patients for eligibility, and the eligible patients will receive written and verbal information about the trial from the research therapists, along with a request to participate.

**Table 1:** Major inclusion and exclusion criteria

**Inclusion criteria**	
1. Over 50 years of age	
2. Had rotator cuff repair surgery	
3. Discharged home after surgery	
**Exclusion criteria**	
1. Previous history of shoulder surgery on the affected shoulder	
2. Severe neurological deficit or infection in the affected shoulder	
3. Severe comorbidities (e.g., uncontrolled diabetes mellitus or rheumatoid arthritis) that will inhibit rehabilitation	
4. Cannot participate in the rehabilitation program	

### Randomization and blinding

Participants will be randomized after the baseline evaluation and allocated to either the Digital or conventional group at a 1:1 ratio. Randomization sequences will be computer-generated blocks, without stratification, by the institution’s biostatistician, who is independent of the study team. The randomized allocation information was sealed in a numbered opaque envelope. The envelope seals will be checked monthly by the principle investigator to make sure the future participants’ allocation is kept blinded. Participant allocation and follow-up arrangements will be managed by the study coordinator, who will not be blinded to the participants’ group assignments. Assessments will be performed by two research therapists from the study team, who will be blinded to the participants’ intervention groups.

### Intervention

#### Conventional group

Participants in this group will undergo a brochure-based home rehabilitation program, which is the standard protocol for patients who had undergone rotator cuff repair surgery in our hospital. The rehabilitation program can be divided into three phases: on-brace, off-brace, and active mobilization. The on-brace phase (immediate post-op period to post-op 6 weeks) mainly consists of low-intensity whole-body exercises with shoulder girdle, elbow, and hand mobilization exercises on the affected side. Participants in this phase are instructed to perform three sets of exercises with 10 repetitions of each set per day. The off-brace phase (post-op 6–9 weeks) consists of passive shoulder ROM exercises using an exercise stick and an early scapular stabilization exercise. The active mobilization phase (post-op 9–12 weeks) consists of both active and passive shoulder ROM and scapular stabilization exercises. During the off-brace and active mobilization phases, patients are instructed to perform 3–5 sets of exercises with 10 repetitions of each set per day. A detailed exercise protocol is described in Table 2.

**Table 2:** Exercise protocols for post-op rehabilitation in patients who had undergone rotator cuff repair

Phase	Exercise		Description
On-brace (immediate post-op period to post-op 6 weeks)	Whole-body exercise	Lunge	
		Trunk rotation	
	Upper extremity mobilization	Finger/wrist/elbow ROM exercises	
		Shoulder shrugging	
Off-brace (post-op 6–9 weeks)	Passive ROM	Forward flexion (supine)	Hold a stick with both hands and move it in both directions using the non-affected arm to passively stretch the affected arm.
		Horizontal abduction (supine)	
		External/internal rotation (supine)	
	Early scapular stabilization	Lower trapezius exercise	Retract and depress the scapula with both hands while holding a towel at both ends.
		Low rowing exercise	Place the palm (affected side) on the wall and try to retract and depress the scapula.
Active mobilization (post-op 9–12 weeks)	Active (assisted) ROM	Forward flexion (supine)	Perform active ROM exercise of the affected arm with help from the unaffected arm.
		Internal rotation (supine)	
		External rotation (standing, 90° abducted)	Gently lean against the wall with the affected shoulder externally rotated in 90° abduction.
		Forward flexion by sliding against a wall	Forward flex the affected arm by sliding along the wall with help from the unaffected arm.
	Scapular stabilization	Rowing exercise	Forward flex the shoulder to 45° and then retract the scapula with a backward pulling motion
		Punching exercise	Retract the scapula by pulling the shoulder backward and punching out straight ahead to protract the scapula.
		Threading exercise	Flex the affected-side elbow to 90° and abduct the shoulder to 45°. Rotate the trunk to the unaffected side keeping the elbow and shoulder angles fixed.
		Lawn-mower exercise	Stand with the feet shoulder-width apart, bend the trunk to the unaffected side. Reach out the affected arm toward the contralateral knee and then return to the normal position by retracting the scapula.

Participants in the conventional group will be educated by a physical therapist, and a brochure with illustrations of the exercises will be provided before they are discharged home. Additional exercise education sessions will be allowed (up to three times, 30 min per session) at the participants’ request.

#### Digital group

The rehabilitation program for participants in this group employs a digital healthcare system. During the on-brace phase, participants in this group will use the same brochure as the conventional group participants for home-based rehabilitation. Participants in the digital group will also be educated by a physical therapist before being discharged home. During the off-brace and active mobilization phases, participants will use the digital healthcare system for home-based rehabilitation. The digital healthcare system consists of four components: software for the rehabilitation program, a three-dimensional (3D)-depth camera (Xbox One Kinect for Windows®, Microsoft, USA), a computer, and a display (TV or monitor). The software consists of an AR-based program for post-op rotator cuff tear rehabilitation, and the 3D camera tracks the participant’s motion until it reaches a pre-determined angle or position. The rehabilitation program and prescribed exercises will be matched with those for the conventional group. Participants in the digital group will use this system at home during post-op 6–12 weeks. The daily exercise amount and progression will be logged by the system, and physicians will review the data at the outpatient clinic. Up to three additional exercise education sessions (30 min each) will also be allowed in this group upon participants’ request.

### Outcomes

#### Primary outcome measure

The primary outcome will be scores on the Simple Shoulder Test (SST), which range from 0 (worst) to 12 (best). The SST is a self-reported questionnaire consisting of 12 dichotomous (yes/no) questions that measure functional limitations [18]. It is a widely used outcome measure for assessing functional improvement in patients who had undergone rotator cuff repair [19, 20].

#### Secondary outcome measures

A number of secondary outcome measures will be employed (Table 3). Pain at rest and during activity will be measured using a numeric rating scale (0–10), and the ROM of the affected shoulder will be measured manually by a research therapist. The shoulder ROM will be measured in degrees in four directions: forward flexion (0–180°), abduction (0–180°), external rotation (0–90°), and internal rotation (0–70°). Muscle strength of the affected shoulder in four directions will be measured by the research therapist using a manual muscle test and rating scale (0–5). Hand grip strength on the affected side will be measured using a Takei handgrip dynamometer (Takei Scientific Instruments Co., Ltd., Tokyo, Japan). Shoulder function will be assessed using the Disabilities of Arm, Shoulder and Hand (DASH) questionnaire and the Shoulder Pain and Disability Index (SPADI). DASH is a 30-item questionnaire that measures the ability of a patient to perform certain upper extremity activities. Scores range from 0 to 100, with a score of 0 indicating no disability and 100 indicating the most severe disability. The SPADI (0–100) consists of a pain scale and a disability scale; higher scores indicate more pain and greater disability. Quality of life will be measured using the European Quality of Life Index (EQ-5D-5L).

**Table 3:** Timetable and measures to be made

	Screening	Post-operation week(s)		
	Baseline	6	12	24
**Primary outcome measure**				
Simple Shoulder Test	X	X	X	X
**Secondary outcome measures**				
Pain at rest and action (NRS)	X	X	X	X
ROM of affected shoulder	X	X	X	X
MMT of affected shoulder	X	X	X	X
Grip strength (JAMAR dynamometer)	X	X	X	X
Function outcomes				
DASH score	X	X	X	X
SPADI score	X	X	X	X
Quality of Life: EQ-5D-5L	X	X	X	X
**Other measures**				
Demographic information	X			
Medical History	X			
Rotator cuff tear size	X			
User satisfaction questionnaires^a^			X	X

*NRS* numeric rating scale, *ROM* range of motion, *MMT* manual motor test, *DASH* disabilities of the arm, shoulder and hand, *SPADI* shoulder pain and disability index, *EQ-5D-5L* European Quality of Life Index

^a^Digital group only

### Sample size

A minimal clinically important difference (MCID) in scores on the SST in patients with rotator cuff tear was reported to be 4.3 [21], and the average score on the SST at post-op 3 months in a previous study was 6.34 (SD = 3.7) [22]. The minimum sample size for the present study was calculated *t*-test based on a 2.15-point difference in SST score (50% of the MCID) and an SD of 3.7, while allowing a 5% probability of two-sided type 1 error and applying 80% statistical power. Forty-nine subjects will be required in each group to allow 15% attrition per group; thus, a total of 115 subjects will be required for the study.

### Data management and sharing

All participant data will be collected by research team members and will be stored in the secured network of Seoul National University Bundang Hospital. Backup database will be updated regularly and only the research team will have access to the database. The case report forms will be stored in the locked cabinet of the research team. The coordinating investigator will have access to the final study dataset and the anonymized dataset will be available on request to the corresponding author. Final results will be published in peer-reviewed journals and presented at conferences, both nationally and internationally after completion of the study.

### Primary hypothesis and data analysis

The primary hypothesis is that participants in the rehabilitation program using a digital healthcare system will demonstrate better shoulder function compared to those in the conventional rehabilitation group. Analyses will be performed using both intention-to-treat and per-protocol approach. The primary outcome measure for this study will be SST scores at post-op 12 weeks. A two-way repeated-measures ANOVA will be used to explore the effects of intervention, time, and intervention by time interaction on SST scores. The results will be presented with a mean difference with 95% confidence interval at each follow-up time point. Continuous secondary outcomes will be analyzed using repeated-measures ANOVA at each post-op 12 weeks and 24 weeks. Subgroup analysis based on the size of the tear will be performed only if there are sufficient participants in all four subgroups (small, <1 cm; medium, 1–3 cm; large, 3–5 cm; massive, ≥5 cm tear or more than two tendons involved) [23]. Statistical analysis will be performed after the 24-week follow-up measurement of the last enrolled participant is done and no inter-rim analysis will be performed during the trial. All tests will use a 5% level of significance (*P* < 0.05).

### Participant safety and withdrawal

The potential risks for participants in this study are minimal. There is no independent oversight committee and adverse events (AEs) will be monitored by the research team at the scheduled follow-ups. Possible AEs include increased pain due to exercise and re-tear of the repaired rotator cuff tendons. Pain will be managed at the orthopedic outpatient clinic with pain medications or steroid injections, if needed. Surveillance of re-tear of repaired tendon will be routinely performed in post-op 12 weeks; however, if the re-tear is clinically suspected at any time during the follow-up periods, appropriate examinations and treatments will be provided. Participants in both groups can report AEs to the research team via direct telephone contact. Participants in the digital group will also receive a telephone number of the manufacturer for technical support.

Participants are allowed to leave the trial at any time. Participants can withdraw from the trial by informing the research team. Participants will be encouraged to visit the pre-scheduled follow-ups even if they withdraw from the study for data collection. Participants in both groups will be regularly reminded with phone calls and text messages to minimize loss of follow-ups and maximize the adherence to the trial.

## Discussion

The prevalence of rotator cuff tears is increasing as the general population ages, which will eventually lead to an increased incidence of rotator cuff repair surgery. Because post-op rehabilitation is a crucial element in restoring function and reducing pain after rotator cuff repair [24], the need for an effective method of post-op rehabilitation is emerging. Post-op rehabilitation of musculoskeletal disorders using VR and AR systems is at an early stage, with only a few published studies showing its feasibility [14, 15, 25, 26].

In this trial, a digital healthcare system will be implemented only during the off-brace (post-op 6–9 weeks) and active mobilization (post-op 9–12 weeks) phases. It will not be applied during the on-brace phase (immediate post-op period to 6 weeks) because exercise programs in this phase are mainly focused on low-intensity whole-body movements under support by a post-operative brace. Also from the previous literatures, patients at this phase have good compliance with seldom having difficulty following the exercise program [27, 28]. The cost of the digital healthcare system (170 USD per week) was also considered. To maximize the cost-effectiveness of rehabilitation using the digital system, we decided that it would only be applied during the off-brace and the active mobilization phases.

This trial would have several strengths. First, to our knowledge, this would be the first randomized controlled trial to compare the effectiveness of post-op rehabilitation using a digital healthcare system with that of conventional rehabilitation in patients who had undergone rotator cuff repair. Second, this trial will implement an AR-based digital healthcare system dedicated to post-op rehabilitation. Some previous studies have used commercial fitness programs such as the Nintendo Wii Fit^TM^ [14, 15], which comprises fitness programs for the general population. However, in this trial, the digital healthcare system is tailor-made for patients who have undergone rotator cuff repair surgery and provides specific exercise programs according to the patient’s rehabilitation phase and daily progress. With this rehabilitation-oriented program, we anticipate that the effectiveness of AR-based post-op rehabilitation will be amplified.

In conclusion, this study will examine the effectiveness of an AR-based digital healthcare system for post-op rehabilitation in the patients after rotator cuff repair. The study will add evidence for the application of digital healthcare systems in post-op rehabilitation.

## Trial status

Protocol ver 3.0 (approved on 2021-08-03)

The study protocol is registered in ClinicalTrials.gov (Identifier: NCT04511377).

Participant recruitment started on 2020-07-30 and is anticipated to be completed by 2022-12-31.

## Supplementary Information

**Additional file 1.** Informed Consent Form (Korean).

**Additional file 2: Supplementary Table 1.** Trial registration data.

## Data Availability

All relevant data from this study will be made available upon study completion.

## Abbreviations

post-op: Post-operative
ROM: Range of motion
VR: Virtual reality
AR: Augmented reality
3D: Three-dimensional
SST: Simple Shoulder Test
DASH: Disabilities of Arm, Shoulder and Hand
SPADI: Shoulder Pain and Disability Index
EQ-5D-5L: European Quality of Life Index
MCID: Minimal clinically important difference
AEs: Adverse events
