# Supplementary material for: Post-operative rehabilitation using a digital healthcare system in patients who had undergone rotator cuff repair: protocol for a single-center randomized controlled trial
Source: Trials. 2022 Aug 17;23:667. doi: 10.1186/s13063-022-06648-4 (PMC9386934; doi:10.1186/s13063-022-06648-4)
Supplement: Supplementary file 1 — Additional file 1. Informed Consent Form (Korean). [file 13063_2022_6648_MOESM1_ESM.pdf]

연구대상자 설명문 및 동의서

대상자 일련번호	

1. 연구 제목

어깨 및 무릎 근골격계 질환 환자에서 실시간상호반응형 디지털 헬스케어시스템을 이용한 병원-가정 연계 단기간 재활치료의 다기관 임상효과 검증

2. 연구책임자

분당서울대병원 재활의학과 임재영 교수

3. 연구의 목적

본 연구는 한국보건산업진흥원의 연구비를 지원 받아 수행하는 연구로 어깨 및 무릎 관절부위 수술을 받은 환자를 대상으로 진단 및 수술 후 유인케어홈시스템을 이용하여 통증이나 관절 기능, 삶의 질에 미치는 영향과 사용 만족도를 확인하고자 하는 연구입니다. 무릎관절 전치환술을 받는 환자 수는 추후 노인 인구 증가에 따라 점차 증가할 것으로 예상되고 있습니다. 무릎관절염은 통증 유발 및 삶의 질 감소, 일상생활동작 및 보행 장애의 원인이 되고 있습니다. 전방십자인대 또한 무릎의 안정성 유지와 스포츠 활동에 매우 중요한 구조로, 스포츠 참여의 활성화에 비례하여 전방십자인대 손상이 증가하고 있습니다. 전방십자인대 수술 후 재활은 수술치료 만큼 중요하며 초기 적절한 재활치료가 제공되지 않으면 수술 후 활동 복귀가 어렵게 될 가능성이 있습니다. 회전근개 질환 또한 이로 인해 일상생활 동작에 제한이 발생할 수 있는 질환으로 재활 운동 치료가 매우 중요합니다. 본 연구에서 적용하는 디지털 시스템을 단기 재활치료가 필요한 근골격계 질환 환자들에게 적용했을 때, 기존의 병원 중심의 재활운동 프로그램보다 관절 기능 및 통증의 호전을 앞당길 수 있는지 알아보고자 합니다. 궁극적으로는 환자들의 삶의 질을 증진시키고, 일상생활 수행 능력의 조기 회복을 통한 보호자의 부담을 경감시키며 의료비를 절감하고 사회 복귀를 촉진시킬 수 있는지 알아보고자 하는 목적의 임상시험입니다.

4. 임상연구 참여대상자 수 및 참여기간

<참여 대상자 수>

- 본 연구에서는 기존의 재활치료와 유인케어 디지털 시스템을 적용하는 재활치료의 효과를 비교하고자 하는 목적으로 기존의 재활치료를 제공 받는 대조군과 유인케어 시스템을 적용 받는 실험군으로 무작위 배정을 통해 진행 할 계획입니다.

무릎관절 전치환술을 시행한 대상자	대조군 15명, 실험군 15명 (총 30명)
전방십자인대 재건술을 시행한 대상자	대조군 15명, 실험군 15명 (총 30명)
회전근개 수술을 시행한 대상자	대조군 50명, 실험군 50명 (총 100명)

<참여 기간> (1회 방문시 1시간 정도 소요)

무릎관절 전치환술을 시행한 대상자	방문 1) 수술 전 입원시<br>방문 2) 수술 후 입원시<br>방문 3) 수술 후 3주<br>방문 4) 수술 후 6주<br>방문 5) 수술 후 3개월<br>방문 6) 수술 후 6개월
전방십자인대 재건술을 시행한 대상자	방문 1) 수술 전 입원시<br>방문 2) 수술 후 입원시<br>방문 3) 수술 후 2주<br>방문 4) 수술 후 6주<br>방문 5) 수술 후 3개월<br>방문 6) 수술 후 6개월
회전근개 수술을 시행한 대상자	방문 1) 수술 전 입원시<br>방문 2) 수술 후 입원시<br>방문 3) 수술 후 6주<br>방문 4) 수술 후 3개월<br>방문 5) 수술 후 6개월

5. 연구의 절차 및 방법

- 대조군에 배정된 대상자들께는 수술 직후 입원 시기에 연구 담당자가 병실에 방문하여 기존의 재활치료대로 브로셔를 배포하여 운동 교육을 시행합니다. 교육 받은 대로 각 가정에서 운동을 하고, 운동일지를 작성하여 방문 시기별 평가를 받습니다.
- 실험군에 배정된 대상자들께는 수술 직후 입원 시기에 연구 담당자가 병실에 방문하여 기존

재활치료를 적용한 브로셔 및 운동일지를 배포하고, 이후에 진행 될 유인케어 홈 장비 설치 관련 안내를 드립니다. 설치일에 맞춰 가정내 장비 설치를 진행하고, 사용법 및 주의 사항을 교육합니다. 장비 설치시에는 1평 공간이 필요하고, 등받이 의자가 필요합니다. 시기별로 운동 수행이 잘 되고 있는지 담당자가 모니터링을 시행하며, 단계를 적용한 운동을 시행한 후에 시기별로 병원에 방문하여 임상 평가를 받습니다.

<무릎관절 전치환술을 시행한 대상자>

대조군 (기존 재활치료)	브로셔 운동 12주 시행<br><br>- 수술 직후에 브로셔를 배포하며 운동교육을 받은 후 수술 후 3개월까지 브로셔 내용의 운동을 합니다.
실험군 (유인케어 적용)	브로셔 운동 3주 + 디지털 헬스 케어 9주 시행<br><br>- 수술 직후에 브로셔를 배포하여 수술 후 3주까지는 대조군과 동일하게 브로셔 운동을 하고, 수술 후 3주부터 수술 후 3개월까지 유인케어 디지털 헬스케어를 이용하여 가정에서 재활운동을 합니다.

- 방문 시기별 하지 기능 평가 시행

(보행속도, 관절가동범위, 하지관절근력, 통증, 하지 기능, 삶의 질, 안전성 평가)

<전방십대인대 재건술을 시행한 대상자>

대조군 (기존 재활치료)	브로셔 운동 12주 시행<br><br>- 수술 직후에 브로셔를 배포하여 수술 후 3개월까지 브로셔 내용의 운동을 합니다.
실험군 (유인케어 적용)	브로셔 2주 + 디지털 헬스 케어 9주 시행<br><br>- 수술 직후에 브로셔를 배포하여 수술 후 2주까지는 대조군과 동일하게 브로셔 운동을 하고, 수술 후 2주부터 수술 후 3개월까지 유인케어 디지털 헬스케어를 이용하여 가정에서 재활운동을 합니다.

- 방문 시기별 하지 기능 평가 시행

(하지 대칭성 지수, 하지 기능, 하지근력, 관절가동범위, 통증, 삶의 질, 안정성 평가)

<회전근개 수술을 시행한 대상자>

대조군 (기존 재활치료)	브로셔 12주 시행<br><br>- 수술 직후에 브로셔를 배포하여 수술 후 3개월까지 브로셔 내용의 운동을 합니다.
실험군 (유인케어 적용)	브로셔 3주 + 디지털 헬스 케어 9주 시행<br><br>- 수술 직후에 브로셔를 배포하여 수술 후 3주까지는 대조군과 동일하게 브로셔 운동을 하고, 수술 후 3주부터 수술 후 3개월까지 유인케어 디지털 헬스케어를 이용하여 가정에서 재활운동을 합니다.

- 방문 시기별 상지 기능 평가 시행

(어깨 기능 평가, 어깨 관절가동범위, 상지 관절 근력, 어깨 통증, 삶의 질, 안정성 평가)

<실험군에게 적용되는 프로그램: 유인케어 홈 (Uincare Home)

1) 성능

(1) 무부착 3D 동작분석 시스템

(2) 25개 관절의 운동동작 실시간 측정

(3) 환자 맞춤형 재활운동

(4) 가정 내 재활운동 수행

(5) 원격 재활운동 결과 확인

(6) 원격 재활운동 스케줄 적용

2) 사용방법

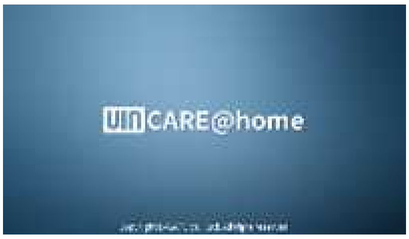

① 전원이 연결되어 있는지 확인하고 컴퓨터를 켜세요.

② 컴퓨터를 부팅 한 후, UINCARE HOME 화면이 표시되는지 확인하세요.

③ UINCARE HOME에 로그인하세요.

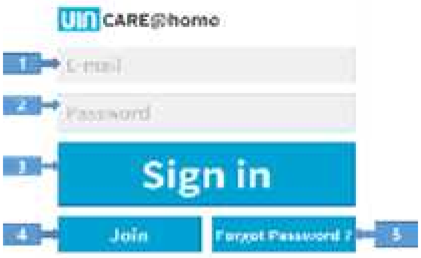

④ 담당 의료진이 정한 오늘의 운동 스케줄을 확인, 선택 및 실행할 수 있다.

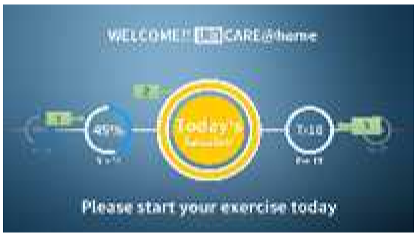

⑤ 프로토콜 정보를 확인하고 실행하세요.

⑥ 자동으로 실행되는 운동 프로그램을 수행하세요.

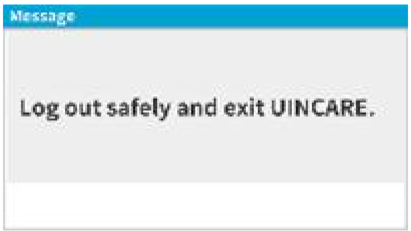

⑦ 수행 후 로그아웃하고 UINCARE 클라이언트를 종료하세요.

3) 신체 인식 방법

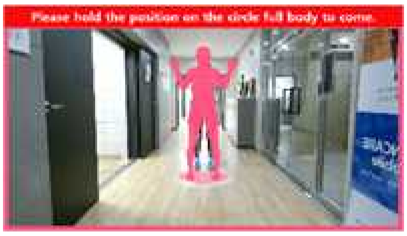

① UINCARE Client 하단의 'Initialize Sensor'버튼을 눌러 화면의 지시에 따라 환자가 인식 영역에 서고 UINCARE가 자동으로 신체 인식을 완료할 때까지 기다린다.

② 신체 인식을 보다 정확하고 빠르게 하기 위해 환자는 UINCARE를 보고 그림과 같이 팔을 곧게 펴고 팔꿈치를 몸 안쪽으로 구부린 다음 팔꿈치를 약 90 ° 구부린 후 양쪽 팔을 들어 올린다.

③ 센서 화면 첫 페이지에서 신체 인식이 제대로 이루어지지 않으면 UNICARE 홈프로그램의 3차원 모션 분석 센서의 렌즈를 2~3초간 가린 후 신체 인식 동작을 반복한다.

④ 신체 인식이 제대로 이루어지지 않으면 잘못된 결과가 발생할 수 있다. 따라서 UINCARE를 사용하기 전에 신체 인식과 관련된 주의 사항을 숙지하도록 한다.

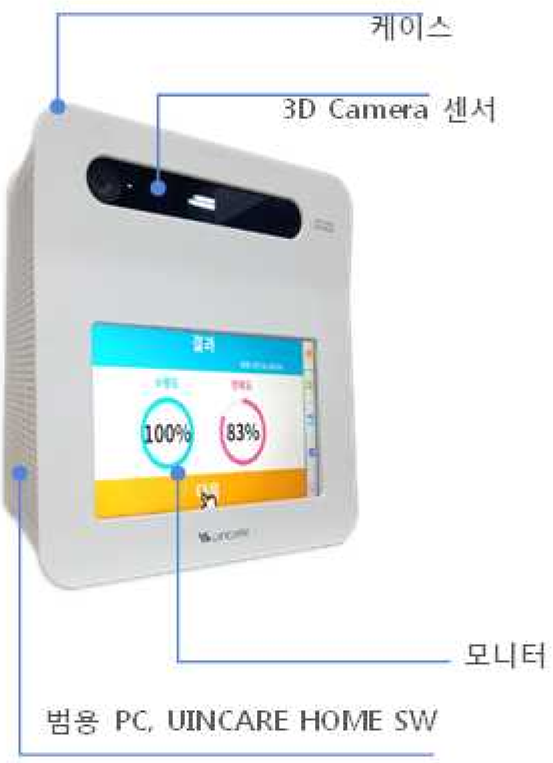

(유인케어 홈플러스 외형) 가로 300mm x 세로 191mm x 높이 332mm, 무게 : 4.5~4.7kg

⑤ Kinect 카메라의 적외선 및 모션 캡쳐 기술을 사용하여 3차원 공간에서 25개의 관절을 추적하여 시행자가 정확한 동작을 수행했는지 판별한다.

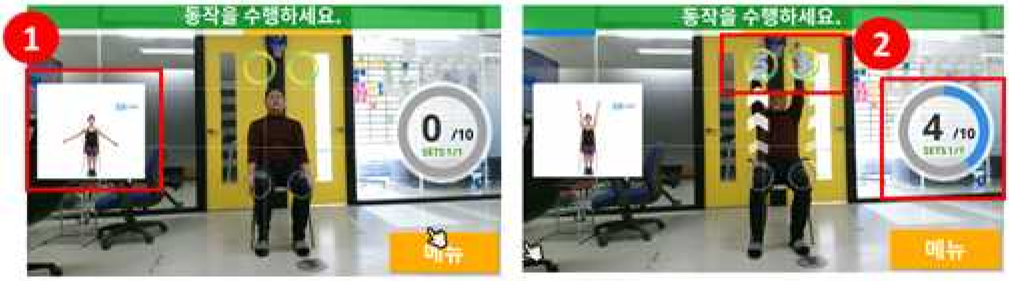

4) 운동 수행

① 화면 안내에 따라 운동을 수행한다.

② 하루에 2-3번, 동작별 8-12회 정도로 1회당 15- 20분 소요, 하루 30-60분 정도 시행한다.

③ 환자는 가이드영상을 보면서 화면의 활동 가이드에 동작을 맞추는 방식으로 진행된다.

④ 실시간 피드백을 통해 올바른 동작 수행 시 운동 횟수가 기록된다.

6. 연구대상자가 준수하여야 할 사항

본 연구에서 실험군에 배정 될 경우 유인케어시스템이라는 기기를 이용하게 됩니다. 기기를 이용하시면서 컴퓨터를 개조하거나 혹은 그 외의 목적으로 분해하거나 개폐해서는 안 되며, 임의로 다른 프로그램을 설치하는 것은 불가합니다. 사용 도중 장치의 전원을 내리거나 강제로 프로그램을 종료시키지 마시고 장치의 문제 발생시에는 담당자에게 연락주시기 바랍니다. 또한 이 장치를 사용하면서 근육통이나 부상 등의 위험이 있을 수 있습니다. 시스템을 사용할 때 통증이나 불편함을 느끼면 시스템 사용을 중단해야 합니다.

7. 연구대상자에게 예견되는 부작용, 위험과 불편함

본 연구에서 적용되는 유인케어시스템은 연구대상자에게 직접적인 접촉이 없으며, 질환별 맞춤 운동 동작을 따라하는 방식으로 운동 치료를 제공하는 시스템입니다. 따라서 연구대상자가 불편을 느끼거나 부작용이 발생하지 않을 것이며, 만약 연구대상자가 불편감을 느끼는 상황이 발생한다면 연구 진행을 즉시 중지하고 연구대상자의 상태를 확인할 것입니다.

8. 연구대상자에게 예견되는 이득

본 연구에 참여함으로써 얻게 되는 직접적인 이득은 없습니다. 하지만 본인의 데이터를 기반으

로 기존의 재활 치료를 종전과 다른 차원으로 발전시켜 유사한 상황의 다른 연구대상자들에게 값진 결과를 제공할 수 있습니다. 또한 연구에 참여하면서 받게 되는 각종 재활 평가에 대한 결과 정보를 받으실 수 있습니다.

9. 연구 참여 비용 및 손실에 대한 보상

본 연구에 참여하시는 분께는 방문시마다 3만원의 참여비 및 교통비를 지급해 드릴 예정입니다. 연구 참여를 중도에 포기하시는 경우에는 1회 내원당 3만원씩 지급해 드릴 예정입니다. 연구에 참여하면서 발생되는 모든 비용은 연구자가 부담할 것이며, 본 연구 참여로 인해 발생하는 피해는 거의 없을 것으로 보이나 만에 하나 발생하는 부작용이 있다면 이에 대한 모든 치료의 경비뿐만 아니라 보상에 대해서 연구 책임자인 임재영 교수가 법적인 책임을 지고 피해보상에 관한 규약에 의거하여 피해 보상할 것이며, 이상반응 및 질환 악화의 경우에는 가능한 한 최선의 치료방법으로 치료할 것입니다.

10. 자발적 참여 및 동의 철회

본 연구의 참여 여부는 자발적으로 결정하실 수 있으며, 언제든지 연구 참여를 중도에 포기하실 수 있습니다. 또한 연구자가 연구의 지속이 어렵다고 판단할 경우에도 연구가 중단될 수 있습니다. 연구참여자가 중도에 연구 참여를 중단하더라도 연구참여자에게 불이익이 있거나 환자로서 치료를 받음에 부정적인 영향이 발생하지 않을 것입니다. 이 연구에 참여하지 않기로 결정하면 현재 본원에서 시행되고 있는 재활 프로그램을 그대로 받게 됩니다. (재활치료실에서 운동 교육 및 브로셔 제공) 이 연구에 참여하기로 결정하면 무작위 배정을 통하여 기존 재활 프로그램을 받게 되거나 유인케어를 이용한 재활 운동 프로그램을 받게 될 것입니다. 또한 연구에 참여하면서 연구대상자로부터 수집한 개인정보는 즉시 폐기할 것입니다.

<연구 참여 도중 대상자의 연구 참여가 중지되는 경우 및 그 사유>

- 우발적인 병발 현상으로 연구의 진행이 불가능하다고 판단되는 경우
- 시험담당자의 판단에 의해 치료 지속이 적합하지 못하다고 판단되는 경우
- 본 과제에서 제공되는 기기 사용이 어려워지는 상황이 발생하거나 평가를 위한 방문이 어렵게 된 경우
- 그 외 시험자의 의견에 따라 임상시험을 진행하는 것이 대상자에게 유익성을 제공하는 것이 아니라고 생각되는 모든 임상적 이상반응 발생시 중도 탈락

11. 개인정보보호 및 개인정보 제공에 관한 사항

본 연구는 개인식별정보를 수집하지 않으며, 본 연구 결과가 출판될 경우 대상자의 신상은 비밀로 보호될 것입니다. 진행 및 종료 후에도 임상연구의 모니터요원, 점검을 실시하는 사람, IRB 및 보건복지부장관이 관계 법령에 따라 연구의 절차와 자료의 품질을 검증하기 위하여 연구참여자의 신상에 관한 비밀이 보호되는 범위에서 대상자의 연구 기록을 열람할 수 있으며, 연구 참여자가 서명한 동의서에 의하여 이러한 자료의 열람이 허용됩니다. 본 연구의 결과는 연구 참여자가 서명한 동의서에 의하여 익명화한 후 데이터 생산을 위한 기초데이터로 제3자에게 제공될 수 있습니다. 본 데이터는 후속 연구, 기록, 축적 등을 위해 3년간 보관할 계획으로 암호가 걸린 외장하드를 이용하여 데이터를 저장합니다.

12. 연구 관련 새로운 정보의 지속적 제공

이 연구가 진행되는 동안에 귀하가 연구 참여 지속 여부를 생각하게 될 만한 새로운 사실이나 정보를 시험자가 알게 되면 언제든지 연구자는 귀하 또는 귀하의 대리인에게 이 사실이나 정보를 알려드릴 것입니다.

13. 담당자 연락처

본 연구에서 연구대상자 권익에 대한 문제, 우려, 질문이 있을 때 상의 할 IRB 또는 임상연구윤리센터 연락처는 아래와 같습니다.

- 책임연구자: 임재영 교수, 분당 서울대병원 재활의학과
  전화번호: 031-787-7732
- 연구담당자: 김보람 연구원, 분당 서울대병원 재활의학과
  전화번호: 031-787-7732

본 연구에서 연구대상자 권익에 대한 문제, 우려, 질문이 있을 때 상의 할 IRB 또는 임상연구윤리센터 연락처는 아래와 같습니다.
만약 연구자로부터 부당한 요청을 받았다고 생각되시거나, 연구과정에 문제점이 있다고 생각되시면, 아래의 윤리심의위원회에 연락하시길 바랍니다.

- 분당서울대학교병원 생명윤리심의위원회(IRB지원실) (전화번호: 031-787-8801~8805)

대상자 일련번호	

연구대상자 동의서

1. 본인은 임상연구에 대해 구두로 설명을 받고 상기 연구 설명문을 읽었으며 담당 연구원과 이 연구에 대하여 충분히 의논하였습니다.
2. 본인은 연구의 위험과 이득에 관하여 들었으며 나의 질문에 만족할 만한 답변을 얻었습니다.
3. 본인은 이 연구에 참여하는 것에 대하여 자발적으로 동의합니다.
4. 본인은 이후의 치료에 영향을 받지 않고 언제든지 연구의 참여를 거부하거나 연구의 참여를 중도에 철회할 수 있고 이러한 결정이 나에게 어떠한 해가 되지 않을 것이라는 것을 알고 있습니다.
5. 본인은 이 설명서 및 동의서에 서명함으로써 의학 연구 목적으로 나의 개인정보가 현행 법률과 규정이 허용하는 범위 내에서 연구자가 수집하고 처리하는데 동의합니다.
6. 본인은 연구 설명문 및 동의서의 사본을 받을 것을 알고 있습니다.

**연구대상자 성명**	**서명**	**날짜(년/월/일)**
**연구자 성명**	**서명**	**날짜(년/월/일)**
