# Supplementary material for: Post-operative rehabilitation using a digital healthcare system in patients who had undergone rotator cuff repair: protocol for a single-center randomized controlled trial
Source: Trials. 2022 Aug 17;23:667. doi: 10.1186/s13063-022-06648-4 (PMC9386934; doi:10.1186/s13063-022-06648-4)
Supplement: Supplementary file 2 — Additional file 2: Supplementary Table 1. Trial registration data. [file 13063_2022_6648_MOESM2_ESM.docx]

**Supplementary Table 1.** Trial registration data

| **Data category** | **Information** |
| --- | --- |
| Primary registry and trial identifying number | ClinicalTrials.gov NCT04511377 |
| Date of registration in primary registry | 13 August, 2020 |
| Source of monetary or material support | Korea Health Industry Development Institute |
| Contact for public queries | Jae-Young Lim, M.D, Ph.D, drlim1@snu.ac.kr |
| Contact for scientific queries | Jae-Young Lim, M.D, Ph.D, Seoul National University Bundang Hospital, Seongnam-si, Korea |
| Public title | Rehabilitation Exercise Using Digital Healthcare System in Patients With Rotator Cuff Repair |
| Scientific title | New Model of Short-term Rehabilitation Exercise Training Using Digital Healthcare System in Patients With Rotator Cuff Repair; Randomized Controlled Study |
| Countries of recruitment | Korea, Republic of |
| Health conditions | Patients who underwent rotator cuff repair surgery |
| Interventions | Active comparator: Conventional Rehabilitation Home-based self-rehabilitation using brochure |
|  | Experimental: Rehabilitation using Digital Healthcare System (Uincare Homeplus) |
| Key inclusion and exclusion criteria | Inclusion: over 50 years of age, had rotator cuff repair surgery, discharged home after surgery |
|  | Exclusion: previous history of shoulder surgery on the affected shoulder, severe neurological deficit or infection in the affected shoulder, severe comorbidities (e.g., uncontrolled diabetes mellitus or  rheumatoid arthritis) that will inhibit rehabilitation, cannot participate in rehabilitation program |
| Study type | Interventional |
|  | Allocation: randomized; interventional model: parallel assignment; Masking: single (outcome assessor) |
|  | Primary purpose: Treatment |
| Date of first enrollment | 30 July, 2020 |
| Target sample size | 115 |
| Recruitment status | Recruiting |
| Primary outcome | Simple Shoulder Test (time frame: 12 weeks) |
| Key secondary outcomes | Pain at rest and action (NRS) (time frame : 12 weeks) |
